# Editorial: Insights in learning and memory: 2022

**DOI:** 10.3389/fnbeh.2024.1399780

**Published:** 2024-04-02

**Authors:** Áine M. Kelly, Denise Manahan-Vaughan, Jee Hyun Kim

**Affiliations:** ^1^Department of Physiology, School of Medicine, Trinity College Institute of Neuroscience, Trinity Biomedical Sciences Institute, Trinity College Dublin, University of Dublin, Dublin, Ireland; ^2^Faculty of Medicine, Ruhr University, Bochum, Germany; ^3^School of Medicine, IMPACT – the Institute for Mental and Physical Health and Clinical Translation, Deakin University, Geelong, VIC, Australia

**Keywords:** human, editorial, behavior, learning, memory

As we approach the second quarter of the 21st century, and investigation into the mechanisms underlying learning and memory continues unabated, fundamental questions on the biological basis of information processing, storage and recall remain unanswered (Ortega-de San Luis and Ryan, 2022). It is necessary in any field of research to make periodic assessments of the state of the art, to outline recent developments and major accomplishments and to identify avenues along which we can move the field forward. In this Research Topic, we explore the new insights, novel developments, current challenges and future perspectives in the field of learning and memory across a number of sub-disciplines. Some of the articles that make up this Research Topic report novel experimental findings, while others review the recent literature published in diverse areas of memory research including fear extinction, food-seeking behavior, and technical and experimental challenges associated with assessing learning and memory function in mice.

The critical role of the amygdala in memory modulation and consolidation is explored in the article by McDonald et al.. They show that amphetamine-induced dendritic changes in nucleus accumbens and prefrontal cortex are preserved in the presence of damage to the amygdala. Given that amphetamine injection results in significant dopamine release in the mesolimbic system, they describe how this experimental approach can provide insights into the role of the amygdala in processing of emotional experiences. The fact that lesions to the amygdala do not prevent the dendritic plastic changes in nucleus accumbens and prefrontal cortex allows the authors to argue against a purely “modulation” view of amygdala function.

In their article, Jiang et al. also use a lesion approach, in this case to investigate the brain regions involved in novel location memory and novel object recognition memory. They demonstrate that lesion to the lateral septal nucleus and the dentate gyrus impairs spatial and object recognition memory respectively, and that the deficit in spatial memory can be rescued by regeneration of neurons subsequent to antisense oligonucleotide injection. The close association of these regions with the hippocampus further highlights the crucial role of the hippocampus in this type of memory processing.

The remaining articles in the Research Topic consolidate and critique recent developments in a range of aspects of learning and memory research. In their discussion of the recent literature on fear extinction, Malik et al. highlight the often-overlooked effect of sex on aspects of learning and behavior. They aim to redress the male-driven imbalance in the literature by drawing together neuroanatomical, neurochemical and behavioral findings uncovered in studies of female mice that collectively show the vital important of sex as a determinant of fear extinction. Furthermore, they demonstrate the relevance of age, from adolescence to older age, to fear extinction in rodents and describe how sex and age interact in the expression of this form of memory. They identify gaps in both the animal and human literature and make recommendations on how these should be addressed in the future in order to make progress in understanding fear extinction across the whole population.

Continuing the theme of translation of findings in animal models to the human sphere, food-seeking behavior in both rodents and humans is the focus of the article contributed by Gladding et al.. In particular, they examine the role of cues in driving excess energy intake and the complex bidirectional relationships between both obesity and highly palatable high-fat/high-sugar diets and cognitive impairments. They focus on rodent and human studies that incorporate Pavlovian-instrumental transfer protocols, describing the neurobiological changes that may underpin dietary effects on the response to Pavlovian cues and proposing the key brain regions and circuits involved. With regard to future experimental directions, they caution the reliance upon BMI as a means of categorizing study participants, arguing that without gathering relevant information on dietary pattern across groups, key information about the relationship between weight, diet and cognition may be overlooked.

Finally, Lang et al. focus on perhaps the most popular and fundamental tool used in experimental learning and memory research in their masterful overview of the development of assessments of cognitive function in the laboratory mouse. They outline the history of the development and use of conventional tests of a variety of forms of memory in the mouse and they delineate recent conceptual and technological advances in learning and memory assessment as a result of AI, virtual reality and automated tracking. The increasing use and biological relevance of experimental settings that control for the effects of the laboratory cage environment on physical activity and social and cognitive enrichment is discussed, along with the importance of efforts to mimic the environment more naturally experienced by the mouse in the wild. They provide suggestions for a roadmap of the future of behavioral assessment in the laboratory mouse that will not only provide better information on learning in the mouse itself, but will enhance the translational potential of findings generated using these techniques.

Learning and memory research is a deep and broad field, as exemplified by the range of papers collected in this Research Topic. However, common themes appear throughout, themes that must be addressed if we are to make the breakthroughs that will direct and shape the next decades of work. These include the key roles of sex, age and lifestyle as biological variables that impact on a range of learning and memory processes and that have often been under-researched in this context, the importance of considering how we best use laboratory animals as experimental tools, and the critical role of technology and artificial intelligence in influencing both experimental design and the interpretation and analysis of experimental findings.

## Author contributions

ÁK: Conceptualization, Writing—original draft. DM-V: Conceptualization, Writing—review & editing. JK: Conceptualization, Writing—review & editing.
